# Hepatitis B assessment without hepatitis B virus DNA quantification: a prospective cohort study in Uganda

**DOI:** 10.1093/trstmh/try117

**Published:** 2018-11-17

**Authors:** Nicolas Laing, Henry Tufton, Emmanuel Ochola, Ojok Godfrey P’Kingston, Mala K Maini, Nicholas Easom

**Affiliations:** 1 St Mary’s Hospital Lacor, Gulu Province, Uganda; 2 Peterborough City Hospital, Edith Cavell Campus, Bretton Gate, Peterborough, UK; 3 Department of Public Health, Gulu University Faculty of Medicine, Gulu, Uganda; 4 University College London, Gower Street, London, UK

**Keywords:** Africa, clinical decision making, health services accessibility, hepatitis B, human viral hepatitis, viral load

## Abstract

**Background:**

Chronic hepatitis B infection affects 240 million people, with the highest prevalence in Africa and Asia, and results in 700 000 deaths annually. Access to diagnostics, particularly for hepatitis B virus viral load quantification (HBV DNA), is a major barrier to treatment. We aimed to test World Health Organization guidelines for hepatitis B management in resource-limited settings.

**Methods:**

We compared treatment allocation with and without the use of HBV DNA in a cohort in Uganda. Hepatitis B surface antigen test–positive, human immunodeficiency virus–negative, treatment-naïve adults were recruited prospectively. Following liver ultrasound and routine haematological and biochemical tests, preliminary allocations into treatment and observation groups were made. HBV DNA was performed for each participant and final treatment decisions were made and compared with preliminary allocations.

**Results:**

Full assessment was completed for 100 participants; treatment was indicated in 20. Assessment without HBV DNA identified patients for treatment with a positive predictive value of 88.2% and a negative predictive value of 94% compared with assessment using HBV DNA.

**Conclusions:**

Where HBV DNA is unavailable, patients with hepatitis B can be assessed by liver ultrasound and routine laboratory tests. These findings will enable physicians in resource-limited settings to initiate treatment more readily and inform policy with regards to viral hepatitis elimination.

## Introduction

Chronic hepatitis B (CHB) affects 240 million people worldwide^1^ and kills around 700 000 people annually through the complications of hepatocellular carcinoma (HCC) and cirrhosis, predominantly in low- and middle-income countries (LMICs).^2,3^ After a long period of neglect,^4^ hepatitis B has become a focus of global health policy. Following the World Health Assembly resolution on viral hepatitis in 2014, the World Health Organization (WHO) published guidelines for CHB in March 2015^5^ and the Global Health Sector Strategy on Viral Hepatitis in June 2016.^6^ These documents describe a goal to eliminate viral hepatitis as a public health threat by 2030, including a 65% reduction in mortality from viral hepatitis, from a starting point in 2016 where <1% of individuals with viral hepatitis currently receive treatment. In the absence of large-scale international funding, the onus is on member countries to establish national guidelines and provide treatment. Therapy with nucleoside reverse-transcriptase inhibitors such as tenofovir controls viral replication,^7^ reverses fibrosis^8^ and reduces the risk of HCC.^9^ These drugs are safe and well tolerated in CHB.^10^

One major barrier to drug treatment for hepatitis B is the cost and availability of investigations that are currently considered key to patient assessment. Quantitative hepatitis B virus (HBV) DNA viral load is performed by real-time polymerase chain reaction (PCR) and non-invasive measures of hepatic fibrosis (e.g. transient elastography) are quantified by devices such as FibroScan (transient elastography). Together with serum alanine transaminase (ALT), these investigations are recommended to inform treatment decisions in European,^11^ American^12^ and Asian^13^ hepatitis B treatment guidelines but are too expensive for many LMIC health care systems. One descriptive study of the attitudes of hepatologists at a conference in West Africa reported the cost and availability of investigations were major reasons these were not performed.^14^ Another qualitative study based on interviews of patients, their relatives and health care workers in Burkina Faso identified access and ability to pay as major barriers to care.^15^ There are no robust alternatives for the developing world and no guidelines for the management of hepatitis B in Africa. The aspartate aminotransferase:platelet ratio index (APRI) was developed for use in hepatitis C infection, where it performs well but has poor sensitivity and specificity in hepatitis B.^16^ The 2015 WHO guidelines^5^ include recommendations for management in settings where HBV DNA quantitation is unavailable, including use of the APRI, but acknowledge research gaps in the indications for initiating or deferring treatment in sub-Saharan Africa. In the absence of funding for large-scale screen-and-treat approaches,^17^ there is a need for simplified clinical assessment of hepatitis B appropriate to LMICs in order to achieve the treatment scale-up required to reduce mortality.^18^

In Uganda, the prevalence of hepatitis B surface antigen (HBsAg) carriage varies from 5% in the capital, Kampala, to 20% in the rural north.^19^ A recent community prevalence survey in Gulu Province demonstrated a prevalence of 17%.^20^ In response to this high prevalence we wanted to establish the efficacy of substituting the use of both HBV DNA and ALT with the use of ALT alone, based on WHO guidelines, as an affordable approach to the assessment of CHB. Accordingly, we designed a pragmatic study, based in the local health care infrastructure, as a real-world test of this approach.

## Methods

### Study design and participants

A single arm prospective cohort study was used to compare algorithms for treatment initiation. Patients were referred to the study by clinical officers and doctors at St Mary’s Hospital Lacor from either outpatient or inpatient services if they had both a positive HBsAg test and a negative human immunodeficiency virus (HIV) test. Referrals were reviewed to ensure they met entry criteria. Individuals whose HIV status was unknown were offered testing. Patients were enrolled only if proven HIV negative. HIV-positive individuals were referred to the local HIV treatment programme for treatment of HIV/HBV co-infection. The study was explained and consent obtained in the local language. A sample size of 103 was calculated to give 80% power to detect a 15% difference between ‘ALT-based’ and ‘with HBV DNA’ assessments by McNemar’s test.

### Laboratory procedures

Clinical assessments were performed by the study clinical officer and reviewed by the study physician. In all patients, full blood count (FBC), liver function tests (LFTs) and serum creatinine (SCr) were measured by the St Mary’s Hospital Lacor clinical laboratory, using a Human Diagnostics 5L automated analyser (Wiesbaden, Germany) and Humastar 600 clinical chemistry platform (Wiesbaden, Germany). Gamma-glutamyl transferase is not a part of the local liver function panel and was not measured. Liver ultrasound was performed and reported by sonographers at St Mary’s Hospital Lacor. Simulated allocation to treatment by an ‘ALT-based’ algorithm (Figure 3) was made at this stage, before viral load results were available. Hepatitis B DNA viral load was measured in each patient by real-time PCR using the COBAS AmpliPrep/COBAS TaqMan HBV test (Roche, Basel, Switzerland).

### Treatment

Treatment decisions were made independently by two authors using the ‘with HBV DNA’ (Figure 2) assessment, discrepancies were discussed and a final management plan was agreed upon. Individuals met treatment criteria if there was evidence of cirrhosis (APRI>2 or ultrasound consistent with cirrhosis) or active hepatitis attributable to HBV (ALT>50 IU/L and HBV DNA>20 000 IU/ml). Patients allocated to treatment were offered tenofovir disoproxil fumarate 300 mg once daily, adjusted according to creatinine clearance^21^ and followed up according to WHO recommendations.^5^ Patients allocated to the observation group were offered follow-up by the hospital outpatient clinic. Tenofovir was donated by Gilead Sciences (Foster City, CA, USA). Gilead Sciences had no input into the study design or data analysis.

### Statistical analysis

‘ALT-based’ assessments were compared with the ‘with HBV DNA’ assessment as a reference standard. Statistical analysis was performed in GraphPad Prism 7 (GraphPad Software, La Jolla, CA, USA). Confidence intervals for proportions were calculated using the Wilson–Brown method.

### Ethics statement

The study protocol was approved by the Lacor Hospital Institutional Research and Ethics Committee and the University College London Research Ethics Committee and registered with the Uganda National Council for Science and Technology. All subjects provided informed consent. No children were recruited into the study. Study information and consent forms were available in English and Luo. All participants were required to give written consent for inclusion in the study.

## Results

**Figure 1.**
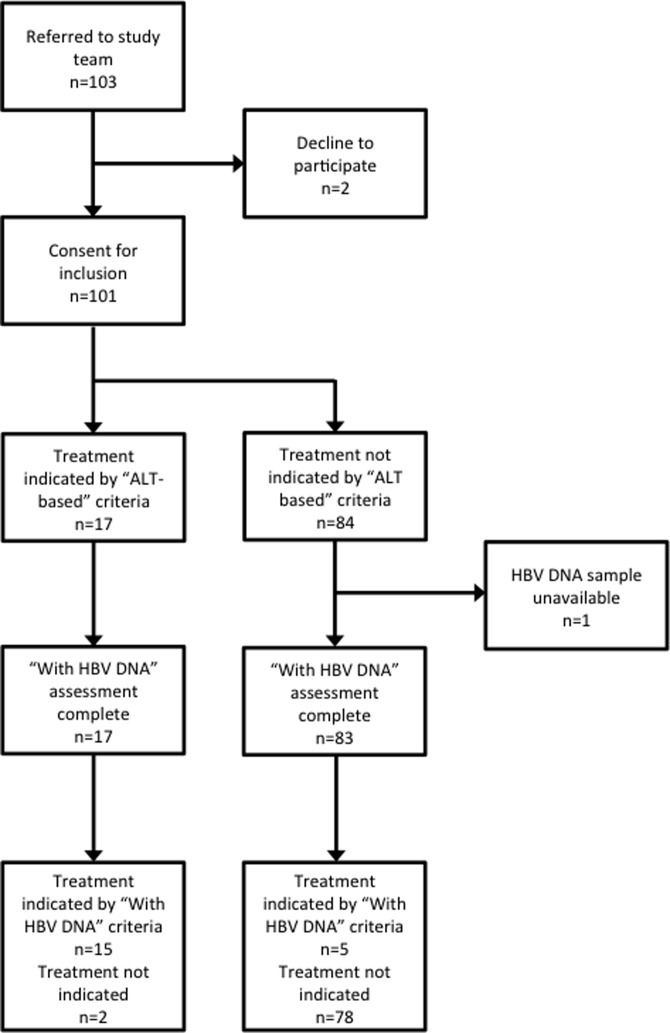
Standards for Reporting Diagnostic Accuracy Studies (STARD) diagram showing comparison of ‘ALT-based’ and ‘with HBV DNA’ algorithms.

Patient flow through the study is shown in Figure 1. A total of 103 patients with positive HBsAg and negative HIV tests were referred to the study from both inpatient and outpatient services. Two individuals declined to participate. A sample for HBV DNA was not obtained from another participant, who was excluded from the analysis. All other patients had full clinical assessment and the following investigations: FBC, LFTs, SCr, liver ultrasound scan and HBV DNA viral load PCR. Demographic and clinical details are summarized in Table 1.

**Table 1.** Summary of demographic and clinical details

Female:male, n	54:46
Age (y), median (range)	27 (18–77)
Outpatient:inpatient, n	96:4
ALT (IU/L), median (range)	31 (11–394)
AST (IU/L), median (range)	37 (8–368)
Platelet count (×10^9^/l), median (range)	212 (56–598)
Creatinine (mg/dl), median (range)	1.0 (0.1–2.7)
HBV DNA viral load (IU/ml), median (range)	652 (not detected–>3.4×10^8^)
Patients with HBV DNA below limit of quantitation, %	20
Patients with HBV DNA>2000 IU/ml, %	42
Patients with HBV DNA>20 000 IU/ml, %	28

**Figure 2.**
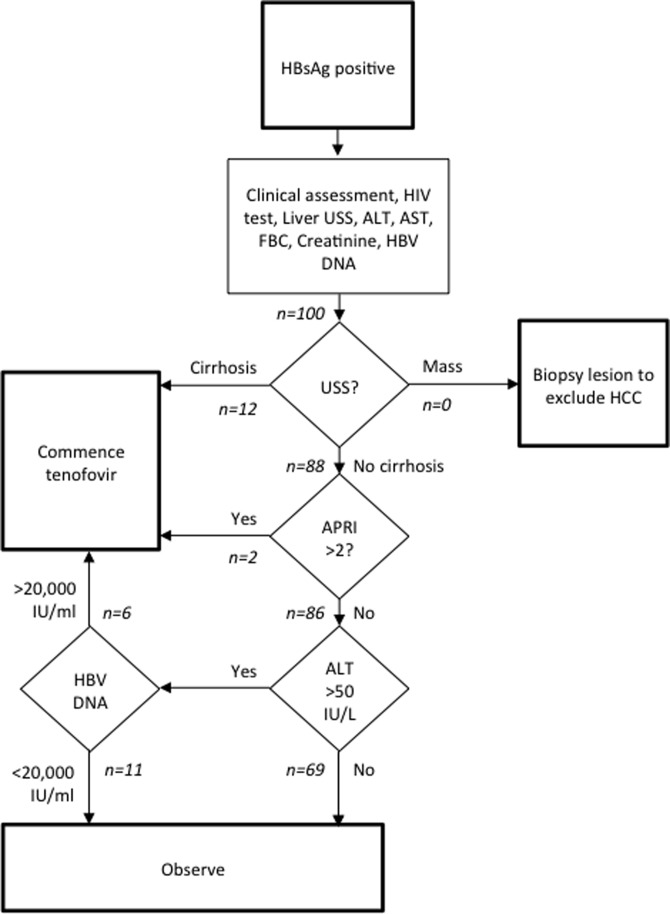
Algorithm for assessment of hepatitis B ‘with HBV DNA’. Based on WHO guidelines for the prevention, care and management of persons with chronic hepatitis B 2015.^5^ Patient flow through the study is indicated in italics. USS: ultrasound scan.

A total of 20 patients met the treatment criteria according to the standard assessment with HBV DNA. Fourteen participants were allocated to treatment due to cirrhosis, 12 with liver ultrasound findings consistent with chronic liver disease, and 2 patients with normal ultrasound scans had an APRI >2. A further six individuals had active hepatitis as an indication for treatment (Figure 2). In total, only eight individuals had an APRI >2. Using treatment criteria of an APRI >2 or both ALT>50 IU/L and HBV DNA>20 000 IU/ml (i.e. without using liver ultrasound), only 14 patients would have met the criteria.

**Figure 3.**
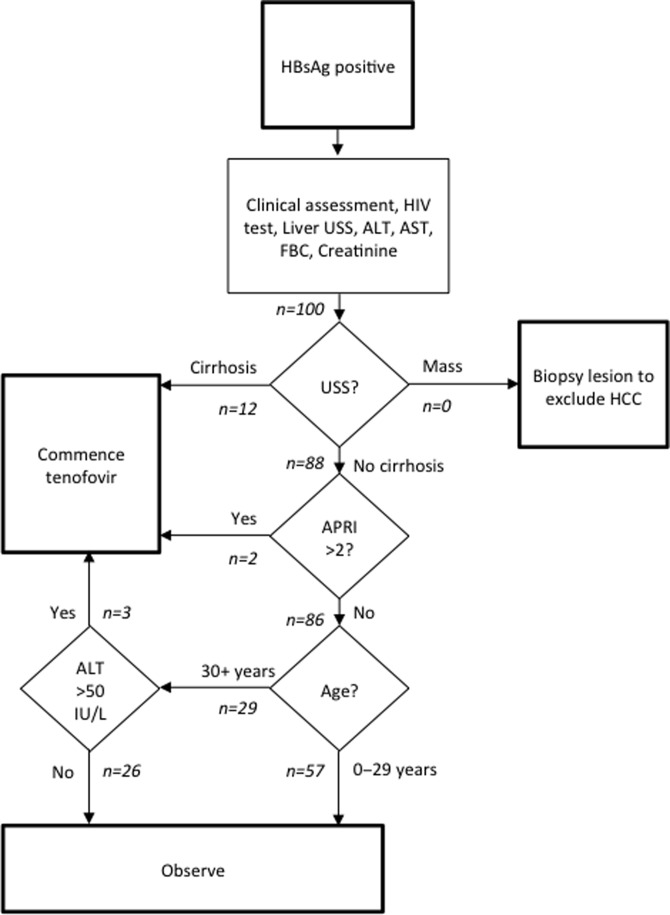
‘ALT-based’ algorithm for assessment of hepatitis B. Based on the WHO guidelines for the prevention, care and management of persons with chronic hepatitis B 2015.^5^ Simulated patient flow by this approach is indicated in italics. USS, ultrasound scan.

We used a single assessment for elevated ALT as a simplified method to identify active hepatitis. Under our ‘ALT-based’ assessment, individuals with cirrhosis and those >30 y of age with ALT>50 IU/L (the upper limit of normal in the local laboratory) were allocated to treatment. Using the ‘ALT-based’ algorithm with no assessment of viral replication, the 14 participants with evidence of cirrhosis were still allocated to treatment, as was 1 of the 6 individuals with active hepatitis. Two of the 81 patients allocated to observation were allocated to treatment by this algorithm (Figure 3). Compared with the ‘with HBV DNA’ approach, this gives a sensitivity of 75% (95% confidence interval [CI] 53.1 to 88.8%) and a specificity of 97.5% (95% CI 91.3 to 99.6%), a positive predictive value (PPV) of 88.2% (95% CI 65.7 to 97.9%) and a negative predictive value (NPV) of 94% (95% CI 86.7 to 97.4%) (Table 2). In a post hoc analysis using any one of cirrhosis on ultrasound, APRI>2 or ALT>50 (in any age) as an indication for treatment, a total of 31 patients were identified (14 with cirrhosis as above, 17 with elevated ALT). This gives 100% (95% CI 83.9 to 100%) sensitivity but 86.3% (95% CI 77.0 to 92.2%) specificity, with an NPV of 100% (95% CI 94.7 to 100%) and a low PPV of 64.5% (95% CI 47.0 to 78.9%).

**Table 2.** Summary of assessment comparison

	Treatment indicated (n=100)	Sensitivity, % (95% CI)	Specificity, % (95% CI)	PPV, % (95% CI)	NPV, % (95% CI)
With HBV DNA	20	–	–	–	–
ALT only	17	75 (53.1 to 88.8)	97.5 (91.3 to 99.6)	88.2 (65.7 to 97.9)	94 (86.7 to 97.4)

The test results of the seven participants with discrepant treatment allocation by the different algorithms are shown in Table 3. There is a tendency for the ‘ALT-based’ treatment strategy to overtreat transaminitis in those >30 y of age and to undertreat young patients with active hepatitis compared with assessment of the same patients using ALT and HBV DNA.

**Table 3.** Test results of seven patients with discrepant treatment allocations

Age (y)	ALT (mg/dl)	APRI	HBV DNA (IU/ml)	With HBV DNA	ALT only
18	94	1.06	109 132 019	Treat	Observe
18	99	1.29	>170 000 000	Treat	Observe
22	67	0.62	>170 000 000	Treat	Observe
26	128	1.77	2 973 114	Treat	Observe
28	135	1.39	198 297	Treat	Observe
32	61	0.37	2910	Observe	Treat
36	51	0.16	310	Observe	Treat

**Figure 4.**
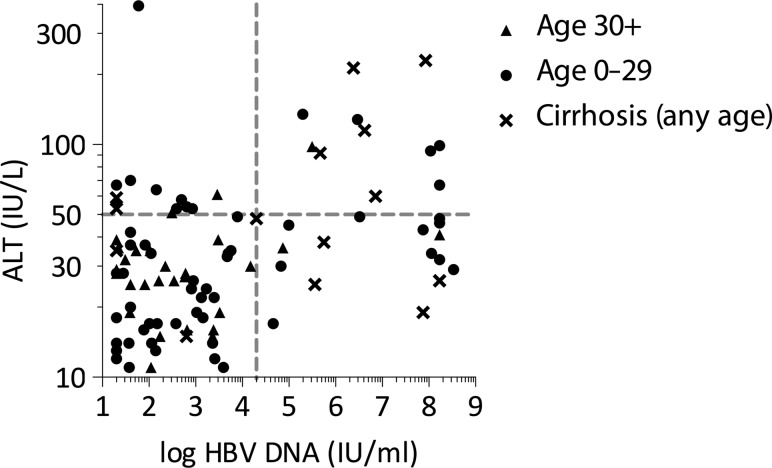
Plot of HBV DNA against ALT. Plot showing log HBV DNA against ALT for individuals with evidence of cirrhosis (APRI>2 or features of cirrhosis on ultrasound, crosses) and those without for ages >30 y and <30 y (triangles and circles, respectively). Horizontal dotted line indicates ALT>50 IU/ml and vertical dotted line indicates HBV DNA>20 000 IU/ml.

ALT and HBV DNA viral loads are shown for the entire cohort in Figure 4. The majority of participants had an ALT <50 IU/L and HBV DNA <20 000 IU/ml (59/100). A group of patients <30 y of age (circles) had high viral loads and ALTs from 30 to 50 IU/L, representing ‘immune tolerance’. Three of 29 patients >30 y of age (triangles) and without evidence of cirrhosis had ALT >50 IU/L, compared with 14/57 patients <30 y of age (p=0.12).

## Discussion

Making treatment decisions is a major barrier to rolling out hepatitis B therapy programs in the developing world. Established treatment guidelines such as from the European Association for the Study of the Liver and the American Association for the Study of Liver Diseases^11–13^ require investigations that are costly for most people. A hepatitis B viral load test in Uganda costs around US$80, where gross domestic product per capita is US$675. As a result, both poor access to assessment and unaffordability of therapy means that millions of people with CHB across the developing world cannot access life-saving treatment. Simplified management strategies based on inexpensive, widely available tests are an important step towards mobilizing large-scale drug treatment programmes. In response to identified research gaps^5^ we have investigated simplified assessment of HBV-infected individuals, based on recommendations from the WHO, that may provide an accessible alternative to established treatment guidelines. In our test site, St Mary’s Hospital Lacor, the cost to patients for all of the tests in the ‘ALT-based’ algorithm is just US$8. The cost of adding HBV DNA quantification to these tests was more than $1140 per altered treatment decision.

Our study demonstrates the identification of a large proportion of CHB patients requiring treatment using only inexpensive investigations that are widely available to physicians across Africa. This finding increases pressure to improve access to effective HBV treatment. The strength of our approach is the use of widely available clinical parameters measured in a real-life health care setting in Uganda. ALT-based assessment is available to physicians in low-resource settings who want to appropriately assess their patients without access to specialist tests. The NPV of the ‘ALT-based’ approach was high, allowing a large number of patients to be confidently reassured. The PPV was high, but the CIs were broad, reflecting both the small number of outcomes and the potential for overtreatment of non-HBV-related transaminitis. This may be particularly relevant in Uganda, where alcohol consumption rates are high. We excluded patients with pre-existing alcoholic liver disease but did not exclude patients on the basis of alcohol consumption, as this is an important cofactor for HBV-associated liver disease. Alcohol consumption is difficult to control for, especially where the alcohol content of locally produced drinks may be variable or unknown. The history may be unreliable and serum ethanol is unlikely to be helpful or feasible. Individuals with significant liver disease and both CHB and significant alcohol intake are advised to reduce their alcohol intake as part of their CHB management.^5^ Co-infection with hepatitis C virus (HCV) may have been a source of non-HBV transaminitis in our cohort, although HCV prevalence in Uganda is low.^22^

Important clinical questions remain. First, the ‘with HBV DNA’ assessment is not a true gold standard against which to compare low-cost alternatives. Clinical assessments including a validated, non-invasive measure of fibrosis such as FibroScan would allow more sensitive detection of early liver disease and provide a more stringent test for any proposed low-cost approach. Second, follow-up data to demonstrate whether a low-cost approach is able to prevent disease outcomes is critical. It may be that a low-cost algorithm that fails to identify a proportion of patients in whom treatment is indicated (according to rich country guidelines) will still be effective if it can detect those at highest risk of progression to decompensated cirrhosis or HCC and prevent these outcomes. Such studies will require large cohorts with long periods of follow-up. Third, the false-positive rate of the ‘ALT-based’ algorithm (2/17) is not negligible. Small-scale overtreatment may be acceptable given the negligible resistance rates of HBV to tenofovir and favourable safety profile both in HBV and in the absence of disease.^23,24^ HIV–HBV co-infected individuals were excluded from this study and referred to the local HIV treatment programme. Under Ugandan national HIV guidelines (and WHO guidelines) they should be treated for both infections with a tenofovir-based regimen without requiring further assessment of liver disease. In HIV-endemic areas it may be possible to use tenofovir as combined HBV treatment and HIV pre-exposure prophylaxis, but this has not yet been investigated. Unlike in HIV, where clinical episodes may be used as treatment triggers, the first presentation of CHB disease is commonly with the lethal complications of HCC or decompensated cirrhosis. As a result, treatment must be commenced in asymptomatic individuals. Treating those in need while minimizing overtreatment will be a challenge for any assessment approach. As we learn more about the consequences of early HBV infection,^25^ we may see broader indications for treatment that simplify assessment, as we have seen with HIV in recent years.^26^

Our findings also emphasize the need for improved tests for fibrosis and active hepatitis, suitable for low-resource settings. Assessment of cirrhosis by liver ultrasound was important, with 12 of the 20 patients allocated to treatment based on this test. To roll out a strategy similar to the one we used in this study, ultrasound provision would have to be improved across rural Africa. In keeping with previous cohorts, APRI had a poor sensitivity for cirrhosis, with 6 of 12 individuals with cirrhosis on ultrasound also having an APRI >2.^27^ By relying on just ALT, APRI and HBV DNA without ultrasound, six individuals with significant liver disease would not have been offered treatment. The gamma-glutamyl transpeptidase (GGT):platelet ratio (GPR), developed by Lemoine et al.^28^ and shown to be superior to the APRI in two cohorts in sub-Saharan Africa, could be incorporated into similar approaches, although measurement of GGT as part of the liver function panel is not universal. We did not have access to GGT locally so could not assess its utility, but this should be the subject of future prospective studies. It is also notable that the proportion of patients allocated to treatment in our hospital-based cohort in Uganda was higher than in a community screened cohort in Gambia reported by the Prevention of Liver Fibrosis and Cancer in Africa investigators (20% vs 4.4%).^17^ The proportion observed in our cohort was more similar to the 32% seen in outpatient settings in the USA,^29^ suggesting that community screening approaches may require assessments with higher specificity than required by a hospital setting. Even in our relatively young cohort, many patients were inactive carriers (44/100), which may keep programmatic treatment costs relatively low, even in high prevalence areas. Nevertheless, 24 of 57 participants <30 y of age had ALT>50 IU/ml (14/57) or HBV DNA>20 000 IU/ml (15/57). Current guidelines emphasize treatment in patients >30 y of age, but CHB in younger individuals may already be inducing premalignant lesions.^25^ Further investigation into the consequences of hepatitis B on the young are important, particularly in sub-Saharan Africa, where the median age is 18 y.

## Conclusions

Inexpensive and accessible assessment such as our ‘ALT-based’ approach should inform trials reporting long-term outcomes as a basis for scale-up of CHB treatment programmes across the developing world. There remains a need to optimize management for CHB in sub-Saharan Africa based on outcome measures, including for young individuals. These issues need to be addressed if the WHO target of a 65% reduction in viral hepatitis deaths by 2030 is to be met. While comprehensive outcome data and improved diagnostic tools are awaited, our pragmatic approach demonstrates that ‘ALT-based’ assessment is a simple, achievable way to identify the majority of patients with CHB who require treatment.
